# The application of nanoparticles in theranostic systems targeting breast cancer stem cells: current progress and future challenges

**DOI:** 10.1186/s13287-023-03584-1

**Published:** 2023-12-10

**Authors:** Xinyu Lin, Ying Wang, Kai Fang, Zijian Guo, Nan Lin, Lihua Li

**Affiliations:** 1 Oncology Institute, Affiliated Hospital of Jiangnan University, Wuxi, 214062 Jiangsu China; 2 Department of Oncological Surgery, Affiliated Hospital of Jiangnan University, Wuxi, 214062 Jiangsu China; 3 Qilu Hospital of Shandong University, Shandong, 250000 China

**Keywords:** Breast cancer, Nanoparticles, Stem cells, mRNA

## Abstract

Breast cancer (BC) is one of the diseases with the highest female mortality rates in the world and is closely related to breast cancer stem cells (BCSCs). Conventional breast cancer chemotherapy drugs target noncancer stem cells (non-CSCs), while cancer stem cells (CSCs) can still survive, which is an important reason for breast cancer drug resistance and local recurrence or distant metastasis. How to eradicate BCSCs while killing BCs is the key factor to improve the effect, and it is also an important scientific problem to be solved urgently. Therefore, targeted BCSC therapy has become a research hotspot. Interestingly, the emergence of nanotechnology provides a new idea for targeting BCSCs. This study summarizes the current application status of nanomaterials in targeting BCSCs, and attempts to construct a new type of lipid nanoparticle (LNP) that can target BCSCs through mRNA, providing a new idea for the treatment of BC.

## Background

The International Agency for Research on Cancer (IARC) released the latest global cancer burden data, showing that the number of new cases of breast cancer (BC) worldwide reached 2.26 million in 2020, making it the common form of cancer in the world. BC is a stem cell disease characterized by the existence of cancer cells with stem-like features and tumor-initiating potential. These cells contribute to tumor dissemination and metastasis [1]. Most chemotherapy regimens for tumors are multidrug combinations. However, common therapies by chemotherapeutic drugs fail to eradicate BCSCs rather than increase the likelihood of recurrence. Currently, there are few well-characterized biomarkers targeting BCSCs, so in recent years some agents targeting BCSCs have been proposed. However, targeted BCSC preparations cannot achieve results in clinical application, and the treatment of BCSCs by nanomaterials has started an enormous wave of research in the medical field. Thus, nanomedicine continues to evolve. From simple nanomaterials to carry drugs to the construction of nanotherapy systems. The construction of nanosystems plays an important role in the diagnosis and treatment of BC.

## The role of BCSCs in the development of BC

### Source of BCSCs

BCSCs were first identified and isolated by Al-Hajj [2] from a patient-derived xenograft model in 2003 and are a small population of BC cells with typical biological characteristics, including self-renewal, multipotent differentiation and tumor initiation. BCSCs play an important role in mediating tumor relapse, metastasis and resistance to chemotherapy or radiotherapy [3]. The origin of BCSCs has been controversial for many years. At present, there are mainly the following views on the source of BCSCs [4]: ① BCSCs are formed by carcinogenic transformation induced by several effective mutations in breast stem cells during quiescence. However, Eun et al. [5] argued that although CSCs share several characteristics with normal stem cells, this does not mean that CSCs originate from malignant stem cells. ② BCSCs were induced by the accumulation of multiple mutations at the level of instantaneous amplified progenitor cells.③CSCs are derived from differentiated cells, and differentiated mammary cells are dedifferentiated to regain self-renewal characteristics and other stem cell-related characteristics. ④ According to the theory of "dislocated somatic stem cells", CSCs may originate from dislocated somatic stem cells. Currently, most researchers believe that BCSCs are derived from breast stem cells and progenitor cells [6]. Recent evidence suggests that changes in the tumor microenvironment contribute to MYC-driven epigenetic reprogramming leading to dedifferentiation of breast epithelial cells into BCSC-like phenotypes [7]. Although many theories have been proposed, there is no concrete evidence for the origin of BCSCs.

### Association of BCSCs with BC recurrence

Although the treatment of BC has made great progress, the mortality rate of BC still ranks first among female malignant tumors, mainly due to the recurrence and metastasis of BC. Data show that the recurrence and metastasis rate of BC is still as high as 23.4%, which is closely related to BCSCs [8–10]. The circulation of BCSCs in the blood also appears in patients after BC surgery. When residual BCSCs are activated by appropriate signals, they often cause tumor recurrence. In addition, studies have shown that BCSCs can promote BC recurrence under the stimulation of some transcription factors and inflammatory factors [11]. Notch‐1 over-activates ERK1/2 through PTEN inhibition, which further activates BCSCs in vivo and in vitro, leading to tumor recurrence [12]. High expression of the CSC regulatory factor CDK12 activates the Wnt or ErbB‐PI3K‐AKT signaling pathway, which induces self-renewal and tumorigenesis of BCSCs, leading to the recurrence of breast cancer [13]. Hong et al. [11] showed that the transcription factor RUXN1 inhibits BCSC activity and directly downregulates the expression of the ZEB1 transcription factor. Segatto et al. [14] found that the fluid discharged from the wound of BC patients after surgery is rich in cytokines and growth factors, which can induce the enrichment of BCSCs with a stem cell-like phenotype through the STAT3 signaling pathway and then cause the recurrence of tumors after surgery.

### Association of BCSCs with BC metastasis

Metastasis is a marker of malignant tumors and one of the main causes of death in BC patients and other cancer patients. BCSCs may be the basis of tumor invasion and metastasis and may promote BC metastasis when special dry markers are highly expressed or under the action of the tumor microenvironment (chemokines, transcription factors and proteins). It is noteworthy that the proliferation and metastasis of BCSCs can also be directly regulated by chemokines, transcription factors and proteins. Studies have shown that the chemokine receptor CXCR3B is significantly upregulated in BCSC subpopulations. Ectopic expression of CXCR3B can increase ALDH activity or the number of CD44^+^ CD24^−^ BCSCs, increase the cell clonogenesis and promote the migration and invasion of cancer cells [15]. Other studies have found that dormant BCSCs regulate the abnormal expression of 6-phosphofructo-2-kinase/fructose-2,6-biphosphatase 3 (Pfkb3) in BCSCs when the expression of autophagy-associated protein 7(ATG7) ATG3 or P62/sequestosome-1 is low, which leads to reactivation of the proliferation program and metastatic growth, promoting BC metastasis [16].

### Association between BCSCs and BC drug resistance

Drug resistance is a long-term problem in the treatment of BC. BCSCs are resistant to chemotherapy and radiotherapy, and conventional chemoradiotherapy can only target BCSCs with active proliferation, but has no killing effect on BCSCs in the resting state. The retention of BCSCs may increase the pool of CSCs in tumors. BCSCs themselves have inherent resistance to neoadjuvant chemotherapy [17]. Studies have found that BC patients with high expression of ALDHs or CD44^+^ cell sets have higher tolerance to chemotherapy, because these cells have the characteristics of BCSCs and are resistant to conventional chemotherapy [18]. Ryoo et al. [19] found that high expression of CD44^+^ led to activation of P62-related NRF2 in CD44^+^ BCSCs, which further promoted the drug resistance of CD44^+^ BCSCs. In addition, the increased expression of ATP-binding cassette (ABC) family transporters in BCSCs may lead to multidrug resistance in tumors [20]. The production of reactive oxygen species (ROS) has long been considered an important mediator of chemotherapy resistance. Recent studies have found that BCSCs can promote drug resistance in BC patients by enhancing ROS [21]. Diedn et al. [22] found radiotherapy resistance of tumor stem cells. Xie Guozhu et al. [23] reported that radiotherapy could enrich tumor stem cells, and BCSCs mediated Her2 subtype transformation and Her2-negative BC cell radioresistance, enhancing radioresistance and aggressivity.

## Application of nanomaterials in treatment of BC

In recent years, nanomaterials have been popularized in the application of tumor therapy due to their special size effect and their ability to target tumor sites through the enhanced permeability and retention (EPR) effect. Nanomaterials can effectively improve the problems of poor bioavailability, low specificity and systemic toxicity of traditional drugs and achieve precise and efficient drug delivery at tumor sites.

Surgical resection is a common and effective method in the treatment of BC [24, 25]. Therefore, the accuracy of surgery is very important, and fluorescent nanoprobes can guide imaging during surgery and improve surgical accuracy [26]. Surgery followed by chemotherapy or radiotherapy is the standard method for cancer therapy including BC. However, due to the side effects that these therapies incur in normal tissues and organs, chemotherapy and radiotherapy usually fail to treat BC. Radiation therapy is a common and effective method for prolonging patient survival and can play a clinical role by inducing DNA damage in cancer cells, leading to DNA damage and ultimately cell death [27, 28]. Moreover, insufficient DNA damage and rapid DNA damage response (DDR) after radiation therapy limit the success rate and efficiency of treatment [29]. Studies have shown that dual DNA-targeting strategies targeting DNA damage and DNA repair can improve the therapeutic effect of radiotherapy [30–32]. In view of the importance of DNA damage and repair in radio-mediated anti-tumor therapy, a team has proposed the use of DNA-targeted nanoparticles to improve the efficacy of radiotherapy. This nanosystem can significantly enhance treatment efficacy, providing a new effective treatment strategy for cancer treatment.

In recent years, various nanoparticles (NPs) have been discovered and synthesized that can selectively target tumor cells without causing any harm to healthy cells or organs. Therefore, NPS-mediated targeted drug delivery systems (DDSs) have become a promising technique for the treatment of BC. Many agents targeting BCSCs have been proposed by researchers, such as targeting BCSC surface markers, inhibiting BCSC-dependent signaling pathways, interfering with BCSC differentiation, targeting metabolism in BCSCs and targeting the breast tumor microenvironment. However, due to poor water solubility, short cycle time, instability and off-target effects, drugs fail in clinical application. BCSC-targeted drug delivery systems can specifically deliver anti-BCSCs drugs to BCSCs without off-target effects by improving bioavailability. In addition, nanodelivery systems can further enhance BCSCs targeting by surface modification of suitable ligands that interact with overexpressed receptors on the BCSCs surface. The delivery strategies of the nanodelivery system for BCSCs mainly include: delivery of anti-BCSC drugs to tumors; combined delivery of chemotherapy drugs and anti-BCSC drugs to the tumor; active targeted delivery of anti-BCSC agents [33]. Wedelolactone, a natural anticancer drug, is ineffective against cancer stem cells. However, Das et al. [34] found that wedelolactone-encapsulated PLGA nanoparticles (nWdl) decreased metastatic potential of BCSCs and enhanced chemosensitivity through coordinated regulation of pluripotent and efflux genes, thereby providing an insight into effective drug delivery specifically for obliterating BCSCs. Moreover, the SAHA/Wnt‐b Catenin antagonist embedded in the gold nanoprotein crown can target BCSCs and inhibit the number of BCSCs [35]. In addition, anti-miRNA delivery using RNA nanoparticles targeting the stem cell marker CD133 [36] and TA6NT‐AKTin‐DOX containing the conjugated DNA nanosequence were targeted to BCSC therapy [37].

At present, researchers pay more attention to nanocarriers, including polymer nanoparticles (PNPs), liposomes and micelles [38]. The advantages and disadvantages of each nanocarrier are summarized in Table 1. It usually consists of three parts: core materials, therapeutic drugs and surface modifiers. Different nanomaterials, such as graphene nanocomposites, gold nanoparticles, Fe_3_O_4_ nanoparticles and polymer composite nanoparticles, play different roles, and the mediated effect of the field is an important mechanism to kill tumor cells and CSCs. The specific process is under the condition of near-infrared irradiation, between tumor cells and cells residing within nanometer carriers. On the one hand, NIR light is absorbed and efficiently converted into heat energy, which can be stored locally to kill tumor cells [39, 40]. On the other hand, the release of high-dose chemotherapy drugs can be slowly controlled to effectively inhibit and kill tumor cells [41].

**Table 1:** Common nanocarriers and their advantages and disadvantages

Carrier material classification	Advantages	Disadvantages
PNPs	Good biocompatibility、biodegradability High therapeutic drug load Easy absorption, control drug release Polymer surface through ligands or targeted modification can achieve multi-functional drug delivery	Easy to bind to negatively charged non-specific cells or proteins High cytotoxicity Low gene transfection efficiency
Liposomes	Good biocompatibility Easy surface modification Wide adaptability to the loaded drugs Long blood circulation time High bioavailability and safety	Long-term application only in small molecule drug delivery Low drug loading rate Poor stability、phospholipids easy oxidation, susceptible to metal Radiation、high temperature、 PH and enzyme effects
Micelle	Enter living cells without the use of transfection agents Long retention time in vivo Good tissue permeability、 biocompatibility、 degradability, Easy structure modification and special "core–shell" structure Uniformity、small volume	Poor physical stability Easy to cause drug leakage and sudden release

### Polymer nanoparticles

Currently, polymer nanoparticles are widely used in drug delivery systems, among which PNPs are one of the most recognized nanoparticles and the simplest soft material that can be used as a nanomedicine [42]. Anticancer drugs can be adsorbed, encapsulated or conjugated within or on the surface of PNPs, enabling the drug to be released to the tumor site over a sustained period of time to dispense the needed dose to the tumor. For instance, the researchers made Bortezomib (BTZ)-loaded (polyethylene glycol) -B -(poly lactic acid), and then, they found that PEG-b-PLA could effectively deliver BTZ to BCSCs, causing the initiation of apoptosis [43]. Sun et al. [44] formulated DOX-tethered gold nanoparticles (DOX-Hyd@AuNPs) and demonstrated that DOX-Hyd@AuNPs could inhibit the growth of BC without increasing the BCSCs subpopulation in the tumor by delivering more DOX into the BCSCs. This can overcome the intrinsic resistance of BCSCs arising from P-glycoprotein drug efflux.

### Lipidosome

Liposomes are colloidal nanocarriers composed of an amphiphilic phospholipid bilayer, which can be loaded with hydrophilic and hydrophobic drugs. Liposomes have the advantages of biocompatibility, easy surface modification and long circulation time in blood, making them ideal nanocarriers for anti-BCSC treatment. Gemcitabine (GEM)-coated hyaluronic acid-coupled liposomes significantly enhance the cytotoxicity, anti-migration, and anti-colony formation of GEM by targeting CD44 expressed on BCSCs, showing promise for the treatment of breast cancer in vitro and in xenograft models targeting BCSC [45]. The greatest advantage of liposomes is that they can achieve passive targeting through the EPR effect [46]. In recent years, immunoliposomes or ligands have been used to target the interaction between liposomes and tumor cells, and antibodies or ligands have been used as guide molecules. Through internalization, large doses of drugs can simultaneously enter the cytoplasm or nucleus of tumor cells, or even suborganelles within the cells, greatly improving the bioavailability of drugs. In addition, gene therapy using liposomes as carriers has increasingly showed advantages in tumor treatment. For RNA interference therapy, a large number of experiments have shown that small interfering RNA (siRNA) can inhibit specific gene expression and show exciting results in vitro. Samson et al. [47] developed glucose-regulated protein 78 (GRP78)-targeted 1,2-dioleoyloxy-3-trimethylammoniumpropane (DOTAP) liposomes to deliver either camptothecin (CPT) and GRP78 siRNA or CPT and clusterin (CLU) siRNA. Both of them exhibited stronger BC cell- and BCSC-targeted activities than free CPT, confirming the synergistic effects of co-delivering anticancer drugs and siRNAs. Therefore, according to the drug resistance mechanism of tumors, gene therapy based on liposome delivery can be used to improve the drug resistance of tumors [48].

### Micelle

Micelles have a hydrophobic core and dozens of nanosize particles on the surface of a water meter and are usually used as carriers of hydrophobic drugs. Polymer micelles are derived from self-assembled amphiphilic block copolymers. These colloidal particles polymerize with hydrophilic and hydrophobic components, providing a platform for a variety of modifications to improve targeting efficiency. Amphiphilic block copolymers are usually composed of two or three blocks, of which PEG is the most common hydrophilic block in the copolymer structure. Polymer micelles have become popular drug carriers for anticancer therapy due to their uniformity, small size and extended circulation time [49]. Paclitaxel encapsulated in micelles has been tested in clinical trials in patients with malignant tumors, which showed reduced toxicity and no change in anti-tumor activity compared to free paclitaxel [50]. Ptx-loaded and anti-CD44^+^ antibody functionalized PLGA-co-PEG polymer micelles have been used in breast cancer cell line therapy. The results show that encapsulation of PTX into the targeted PLGA-co-PEG micelles increases the sensitivity of BCSCs to PTX [51]. Zhang et al. [52] demonstrated that co-loaded micelles loaded with the BCSC inhibitor staurosporine (STS) and the cytotoxic drug Epirubicin (EPI) can inhibit breast cancer cells and BCSC-associated subgroups (such as ALDH + and CD44 + /CD24 − subgroups). The STS/Epi-loaded micelles (STS/Epi/m) demonstrated potent therapeutic efficacy against both naïve orthotopic 4T1-luc breast tumors and their recurrent Epi-resistant counterparts, significantly prolonging survival.

## Lipid nanoparticles (LNPs)

LNPs are essentially tiny lipid vesicles, typically assembled from synthetic lipids and other molecules such as cholesterol, phospholipids and polyethylene glycol. The core component, synthetic lipids, is usually composed of a hydrophilic head structure, a hydrophobic lipid tail structure and a linker linking the head and tail. LNPs are widely used in various fields because of their targeting function, such as targeting the precise site of radiotherapy and chemotherapy, and mixing drugs for precise delivery. LNPs are biocompatible vehicles of phospholipid monolayer, structures that can wrap mRNA in lipid nuclei and avoid degradation. The latest study found that mRNA drugs delivered by LNPs may become an effective means for the treatment of LAM, a serious lung disease [53]. Hence, we speculated that mRNA drugs delivered by LNPs may become an effective means for the treatment of BCSCs. We can build a new LNP system that can target BCSCs by carrying mRNA and mixing hyaluronic acid, fluorescent dyes, etc., which may solve the problem of targeting BCSCs and provide new targeted treatment technology for BC.

### Role of LNPs in nucleic acid drug delivery

Nucleic acid drugs include antisense nucleic acid (ASO), small interfering RNA (siRNA), microRNA (miRNA), small living RNA (slRNA), messenger RNA (mRNA) aptamer, ribozyme and ARC. As a form of gene therapy, nucleic acid drugs can be made into new drugs simply by rearranging the sequences of A, G, C and T(U). Nucleic acid drugs can target molecules that cannot be targeted by chemical drugs or antibody drugs (such as mRNA and miRNA), which is expected to produce breakthroughs in diseases where traditional drugs have poor efficacy [54]. Due to circumstances caused by the COVID-19 pandemic, mRNA vaccines and nucleic acid drugs have attracted increasing attention. Currently, most mRNA vaccines [55, 56] and nucleic acid drugs use LNPs to deliver mRNA and other nucleic acid molecules. Compared with other types of nucleic acid drug delivery systems, LNPs are more conducive to drug delivery due to their high nucleic acid encapsulation rate, effective cell transfection, strong tissue penetration, low cytotoxicity and immunogenicity [57].

#### Main problems in the development of nucleic acid drugs

Nucleic acid drug development also faces three major problems. First, nucleic acid molecules in the body are not stable, nucleic acid drug molecules, regardless of their base composition, structure and endogenous nucleic acid molecules, are vulnerable to all kinds of nucleic acid enzyme degradation, and their RNA—phosphate skeleton structure is electronegative, polar, and completely unable to meet the basic conditions of drug use [58, 59]. There is a contradiction between high design efficiency and a low proportion of drugs in nucleic acid drug research and development. The second, nucleic acid molecule has potential side effects, mainly including hepatotoxicity and immunotoxicity. This toxicity mainly comes from two aspects. One is sequence-dependent toxicity. Some specific segments, such as CpG, 5′-UGUGU-3′, 5′-GU-CCUUCAA-3′, can interact with Toll-like receptor (TLR) proteins to induce the production of cytokines and chemokines. The activation of nucleic acid fragments to the complement system produces toxicity [60]. The second is the off-target effect based on mismatch. The most important problem is that the development of nucleic acid drug delivery systems is difficult. How to ensure that there is enough time for nucleic acid drugs precisely target the lesion site, and avoid damage to normal cells after the drugs are injected into the body, must be investigated for the development of efficient and safe drug delivery systems to solve the problems of nucleic acid drug delivery, stability, and off-target effects, LNPs are currently used in nucleic acid drug research as a delivery system and are one of the most safest and most effective delivery methods of delivering nucleic acids.

#### How to use LNPs to solve the problem of nucleic acid drugs

RNAi (RNA interference) is an important method of targeted therapy, but RNAi transfection efficiency is low, and RNA is easily degraded by intracellular nucleases [61]. The nanoparticle carrier is highly efficient and nontoxic and has high transfection efficiency. Lipoid-polycationic-nucleic acid complex nanoparticle carriers (LPNs) have been a hot topic in recent studies. Polycations condense nucleic acid molecules into nanoparticles, and polyethylenimine (PEI) is a polycationic carrier with abundant cationic charge, which makes it easier for genes to enter cells after condensation. Liposomes are wrapped in RNA viruses and compressed into LPN structures. The core is composed of a nucleic acid or other genetic material, a lipid shell to protect the nucleic acid and the kernel from other nuclease degradation and differentiation, because of similar lipid shell and cell membrane structures, by swallowing the cell whole or entering the cell to gobble up the target gene, prompting LPN and cell membrane fusion and completing the transfection.

## Composition and key excipients of nucleic acid-delivered LNP

### LNPs composition

**Fig. 1:**
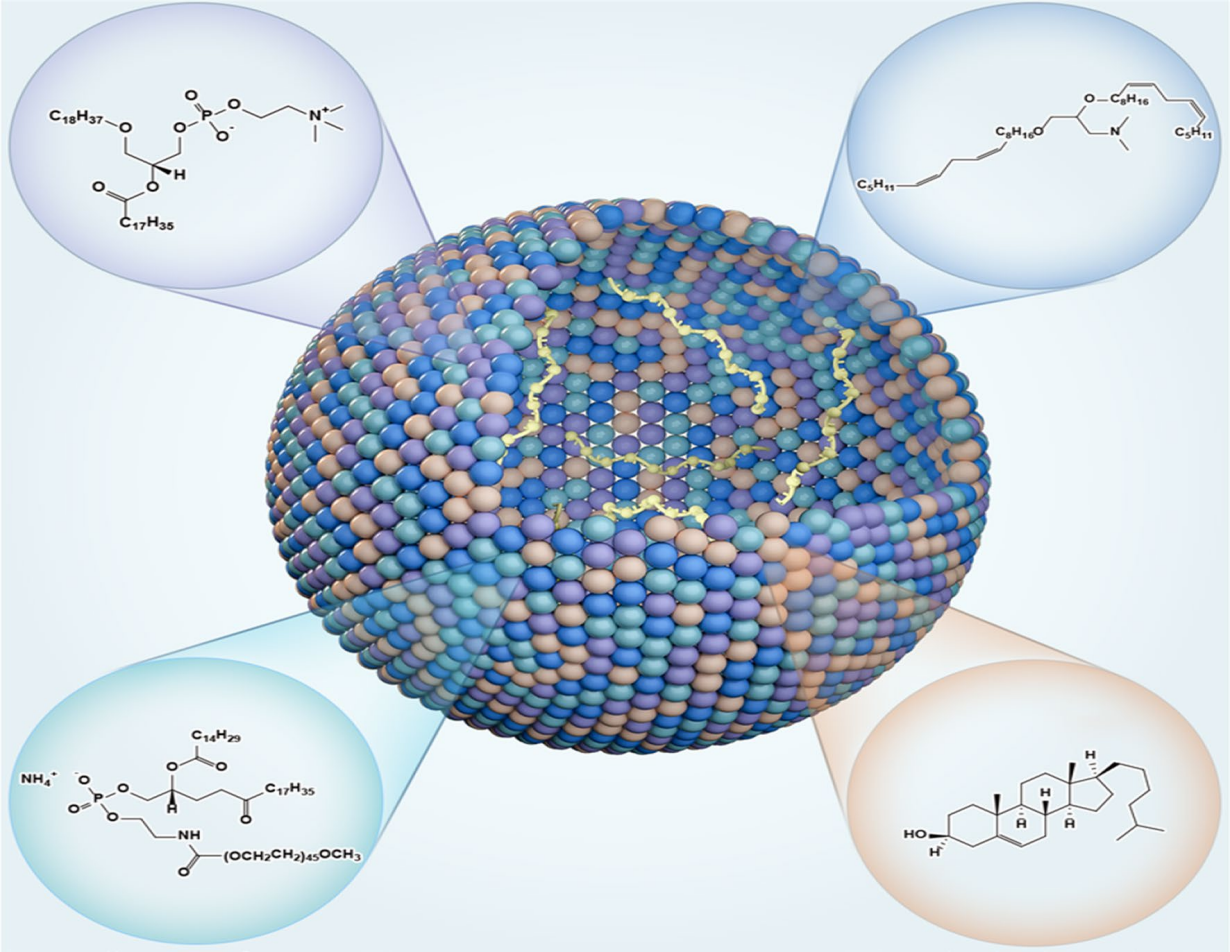
The composition of nanoparticles

**Fig. 2:**
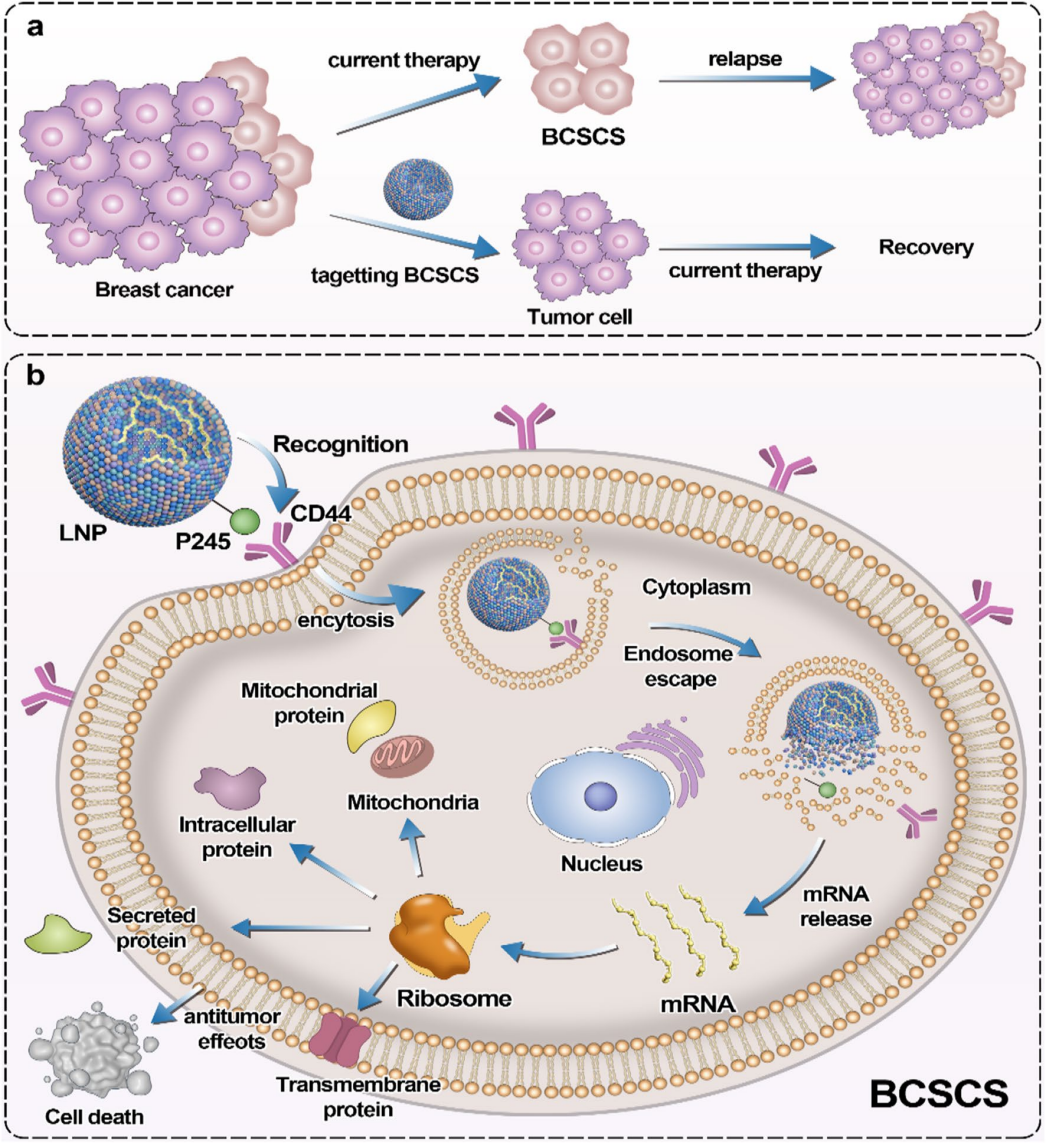
Application of targeted breast cancer stem cells. The difference between general therapy and targeted BCSCS (**a**). LNPs carry mRNA target BCSCs (**b**)

Some companies have very similar lipid ratios for nucleic acid delivery systems, with 50% ionizable cationic lipids in LNPs, 10% neutral lipids and 38–40% cholesterol. LNPs mimic membrane-forming cells and are only approximately 100 nm in diameter, consisting of neutral lipids, cationic lipids, cholesterol and PEG lipids. The specific molecular composition of LNPs is shown in Fig. 1. Compared with common chemical drugs, nucleic acid drugs have a large number of phosphoric acid radicals, which can be negatively charged, thus making ionizable cationic lipid LNPs better able to wrap nucleic acid drugs [62]. Cationic lipids have a hydrophilic end with ammonium silver, which is positively charged when combined with hydrogen ions under acidic conditions. Nucleic acids can be wrapped in lipid nanoparticles by electrostatic adsorption of the two. When LNPs are enucleated, ionizable cationic lipids ionize in acidic environments and destroy the endosomal membrane, thereby achieving inclusion escape [63]. Conventional therapeutic agents mainly kill the bulk of breast tumor cells and fail to eliminate BCSCs, even enhancing the fraction of BCSCs in breast tumor (see Fig. 2a). LNP may be designed as a specific binding carrier lipoprotein. LNPs may bind to receptors on the surface of breast cancer stem cells and release drugs through endocytosis to target BCSCs. The wrapped LNP shell shows hydrophobicity due to the outward hydrophobic end of cationic lipids. At this point, PEG lipids, which are commonly used in the synthesis of traditional liposomes, can be added to make the hydrophobic end of PEG lipids combine with the hydrophobic end of cationic lipids, while the hydrophilic end of PEG lipids (with PEG) forms the outer shell of nucleic acid lipid nanoparticles [64]. At the same time, cholesterol can be added to improve the stability of nucleic acid lipid nanoparticles, so that the hydrophobic end of PEG lipids and the hydrophobic end of cationic lipids can be more closely combined, and the finished product of nucleic acid lipid nanoparticles can be obtained [65].

### Key excipients for LNPs

1. Cationic lipids combined with negatively charged mRNA can efficiently package nucleic acid drugs, while providing positive charge, and negatively charged mRNA compound, cations escape, mRNA transfection in vivo, and ionized lipids have PH sensitivity.
2. Auxiliary lipids: can stabilize particles, destroy the stability of the contents and improve the efficiency of nucleic acid delivery.
3. Cholesterol: stabilizes the LNP structure, regulates membrane fluidity and improves particle stability.
4. Pegylated phospholipids: improve particle stability, reduce particle binding with plasma protein in vivo and prolong systemic circulation time.
5. Stabilizers sucrose or trehalose can improve the stability of LNP and mRNA vaccines and prevent excessive lipid viscosity.

## Novel LNPs target BCSCs via mRNA

For the past few years, increasing interest has been seen in using the LNPs to deliver mRNA therapeutics. The in vitro synthetic mRNA has the potential to produce therapeutically relevant proteins “in vivo” to control and treat a broad spectrum of diseases, including AIDS, rare diseases, cancer, and coronavirus. A research team developed a lung-targeted LNP that specifically delivered the mRNA encoding the normal Tsc2 gene to TSC2-deficient TTJ cells for the treatment of lymphangitic pulmonary fibroids (LAM) caused by Tsc2 gene mutation [53]. Yulia Rybakova et al. [66] used LNP to deliver trastuzumab encoded mRNA to treat tumor-bearing mice, selectively reducing the volume of HER2-positive tumors and improving the survival rate of animals. Compared with direct injection of trastuzumab protein, the pharmacokinetic characteristics of the protein produced by LNP injection were improved.

CD44 is specifically overexpressed in BCSCs and is a specific marker for BCSCs [67]. P245, an anti-CD44 antibody, has been demonstrated to inhibit BC growth and eliminate BCSCs in xenograft mouse models [68]. We attempted to construct an LNP containing mRNA encoding a candidate anti-tumor protein. LNP coupled to P245 can bind the CD44 receptor to target BCSCs. LNPs traps mRNA in the core through microfluid-controlled preparation method and delivers mRNA-targeted to cells through endocytosis and lysosomal escape pathway, thus synthesizing anti-tumor proteins in vivo and playing anti-tumor effects (see Fig. 2b). At the same time, we can add fluorescent dye as a tracer, which can carry out targeted therapy more accurately.

## Conclusions and future directions

Currently, there are still many problems in the targeted application of novel LNPs to BCSCs. Several studies have been able to combine the CIRSP-Cas9 gene editing system with LNPs for targeted delivery to the lungs, so we can apply gene editing technology to BC and combine gene editing technology with new LNP systems. It is possible to develop an LNP delivery system targeting BCSCs, and complete technological transformation to transform killer tumor cells into a targeted treatment of BCSCs, to reduce the recurrence and metastasis of BC. The innovation of this paper lies in the novel LNP system we constructed, which carries mRNA and adds fluorescent dye to trace, providing a new idea for the early identification and targeted treatment of BCSCs.

## Data Availability

Data sharing was not applicable to this article as no new data were created or analyzed in this study.

## Abbreviations

BC: Breast cancer
BCSCs: Breast cancer stem cells
IARC: The International Agency for Research on Cancer
non-CSCs: Non-cancer stem cells
LNP: Lipid nanoparticles
ALDH1: Aldehyde dehydrogenase 1
ABC: ATP Binding Cassette
ROS: Reactive oxygen species
EPR: Enhanced permeability and retention
DDR: DNA damage response
NPs: Nanoparticles
DDS: Drug delivery system
PLGA: Polylactide glycolide
DOX: Doxorubicin
ASD-BSA: AUN@C16H32O2S-DOX-BSA
LNPs: Lipid nanoparticles
siRNA: Small nuclear interfering RNA
ASO: Antisense nucleic acid
miRNA: MicroRNA
SLRNA: Small living RNA
mRNA: Messenger RNA
TLR: Toll-like receptor
PEI: Polyethylenimine
